# Low practical knowledge but high willingness to use malaria rapid diagnostic tests among healthcare workers in the Republic of Congo

**DOI:** 10.1186/s12889-026-28761-8

**Published:** 2026-08-11

**Authors:** Marcel Tapsou Baina, Alain Maxime Mouanga, Christevy Vouvoungui, Steve Diafouka-Kietela, Marliti Ngambou Nguissaliki, Nafissatou Diallo, Francine Ntoumi

**Affiliations:** 1 Fondation Congolaise pour la Recherche Médicale, Brazzaville, Republic of Congo; 2 Faculty of Sciences and Techniques, Marien Ngouabi University, Brazzaville, Republic of Congo; 3 Faculty of Health Sciences, Marien Ngouabi University, Brazzaville, Republic of Congo; 4 Société Congolaise de Santé Mentale, SOCOSAM, Brazzaville, Republic of Congo; 5 Ministry of Public Health and Population, Brazzaville, Republic of Congo; 6 Institute of Tropical Medicine, University of Tübingen, Tübingen, Germany

**Keywords:** Malaria, Rapid diagnostic tests, Knowledge, Attitudes, Practices, Healthcare workers, Republic of Congo

## Abstract

**Background:**

Malaria rapid diagnostic tests (mRDTs) are essential for early detection and effective management of malaria, but their success depends on healthcare workers’ (HCWs) knowledge, attitudes, and practices regarding their use. This study described these factors and aimed to identify the key barriers that must be addressed to improve diagnostic malaria practices in the country.

**Methods:**

A descriptive cross-sectional study was conducted from November 2024 to February 2025 across 09 health districts in Brazzaville department. Using a two-stage random sampling strategy, 36 public and private primary healthcare facilities were selected, with 211 HCWs involved in malaria diagnosis were enrolled. Data were collected through, a pre-tested semi-structured questionnaire that was self-administered by participants in the presence of the data collector, who provided clarification when participants encountered difficulties understanding certain items, and were analysed using R software (version 4.2.3). Group comparisons of proportions were performed using the Chi-square test (or Fisher’s exact test when expected cell frequencies were below 5. Knowledge, attitude and practice scores were computed from predefined items and classified as good (≥ 75%), intermediate (50–74.9%), or insufficient (< 50%).

**Results:**

Among participants, 96% reported using mRDTs, though only 36% had received specific training. General malaria knowledge was low, with just 9% scoring good. Most (90%) had poor understanding of target antigens and diagnostic procedures. However, 97% and 59% perceived mRDTs as easy to use and reliable, respectively. In practice, 85.8% used both mRDTs and microscopy, but only 7.6% followed national guidelines. Following negative mRDTs in symptomatic patients, 39% requested microscopy, and 27% prescribed antimalarials based on clinical suspicion. No significant associations were found between knowledge, attitudes or practices and demographic or professional characteristics.

**Conclusion:**

Although mRDTs usage is widespread among HCWs in Brazzaville department, significant gaps persist in their knowledge, attitudes, and practices regarding national diagnostic protocols. Enhanced training and policy enforcement are needed to strengthen malaria case management in the Republic of Congo.

**Supplementary Information:**

The online version contains supplementary material available at 10.1186/s12889-026-28761-8.

## Background

Malaria remains a leading cause of morbidity and mortality in tropical regions. According to the 2024World Health Organization (WHO) World Malaria Report, presenting data for 2023, sub-Saharan Africa accounted for 94% of global malaria cases and 95% of malaria-related deaths, with Central Africa alone reporting approximately 60 million cases and 124,000 deaths in 2023 [1]. In the Republic of Congo, where the present study was conducted, is part of this Central African sub-region and contributes directly to this regional burden, with malaria constituting the primary cause of medical consultations (59%), hospital admissions (61%), and mortality (22%) in the country [2].

Effective malaria control relies on timely and accurate diagnosis. The gold standard remains the microscopic examination of Giemsa-stained thick blood smears [3]. However, the sensitivity of microscopy depends largely on the skill of the microscopist and the quality of the laboratory equipment [4]. Although molecular techniques such as polymerase chain reaction (PCR) offer superior sensitivity detecting as few as 1 to 5 parasites/µL of blood [5], they are expensive and require additional trained personnel, which limits their routine use in low-resource settings.

In this context, malaria rapid diagnostic tests (mRDTs) have emerged as a practical alternative due to their ease of use, minimal infrastructure requirements, and rapid turnaround time. These tests detect specific *Plasmodium* antigens such as histidine-rich protein 2 (PfHRP2), *Plasmodium* lactate dehydrogenase (pLDH), or aldolase. However, the persistence of some antigens in the bloodstream after parasite clearance can result in false-positive results [6–8]. Despite this limitation, mRDTs remain essential for malaria diagnosis, particularly in low- and middle-income countries where access to microscopy and stable electricity is limited.

In the Republic of Congo, the National Malaria Control Strategy (2023–2027) aimed to ensure quality parasitological diagnosis for 90% of suspected malaria cases nationwide [9]. However, only 72% of suspected cases were tested using microscopy or mRDTs, likely due to a lack of diagnostic tools and gaps in HCWs’ diagnostic capacity [2]. Previously, the country maintained a sufficient number of well-trained microscopists, but their numbers have declined due to reduced training opportunities and a shortage of functional microscopes in health facilities [10]. As malaria control and elimination efforts increasingly depend on mRDTs, their effectiveness relies heavily on the attitudes of HCWs, which are shaped by their knowledge and willingness to use these tests. Understanding how knowledge and attitude influence diagnostic practices is crucial to identify operational barriers and design targeted interventions to strengthen malaria diagnosis and case management in the Republic of Congo.

This study aimed to describe HCWs’ knowledge, attitudes, and practices regarding mRDTs use and to identify the key barriers that must be addressed to improve malaria diagnostic practices in the country.

## Methods

### Study design

A cross-sectional descriptive study was conducted between November 2024 and February 2025. The study used a quantitative approach based on a semi-structured questionnaire administered to HCWs operating in both public and private healthcare facilities.

### Study setting and sampling strategy

The study took place in 9 health districts of the Brazzaville department: Makélékélé, Bacongo, Poto-Poto, Moungali, Talangaï, Mfilou, Djiri, Madibou, and Ouenzé. At the time the study was designed, Brazzaville had a total of 52 health centers across these 9 health districts, according to the district health authorities. Only first-level (primary) healthcare facilities were included, providing general outpatient consultations and malaria diagnosis (via mRDTs and/or microscopy), but not inpatient or specialised hospital-level care. Facilities had to be operational, accessible, and must have performed mRDTs in the six months preceding the survey. Brazzaville was selected as the study setting because it represents a high malaria caseload area for the country [2]. A two-stage stratified random sampling strategy was employed. First, a simple random sampling approach was used to select primary healthcare facilities. A complete list of all first-level facilities was obtained from district health authorities, and four eligible healthcare facilities were randomly selected per district, yielding a total of 36 facilities. Second, within each selected healthcare facility, HCWs involved in malaria diagnosis were sampled using simple random selection, with at least four HCWs recruited per facility. Eligible participants were HCWs (Physicians, nurses, midwives, laboratory technicians, and other healthcare personnel) who had been providing malaria diagnosis or treatment services for at least six months at the time of the survey, and who provided written informed consent. HCWs were excluded if they were on extended leave, unavailable during the study period, or declined to participate. The recruitment period lasted from 19 November 2024 to 14 February 2025.

### Data collection

A semi-structured questionnaire was developed based on a review of KAP tools used in comparable settings [11, 12] to capture sociodemographic information and data related to knowledge, attitudes, and practices regarding the use of mRDTs. Sociodemographic variables collected included sex, age, professional category, years of experience, health district, type of facility (public/private), and additional training in infectious diseases or diagnostics. Prior to implementation, the questionnaire was pretested with 10 HCWs not included in the final sample to assess clarity, and the average time required for completion. Based on feedback, modifications were made to improve question wording and structure.

The final version was administered in French, the official language of the healthcare system in the Republic of Congo. Data were collected through a pre-tested, semi-structured questionnaire that was self-administered by participants during in-person sessions, in the presence of a data collector who provided clarification when participants encountered difficulties understanding certain items. Data collectors were researchers already familiar with malaria diagnosis and the study questionnaire; no separate, formal training module specifically on responding to participant questions was organised, but their professional background allowed them to clarify terminology or rephrase questions when participants requested help, without suggesting a particular answer. Responses were initially recorded on paper forms and subsequently digitized using the KoboCollect platform. Each interview lasted between 45 and 60 min. Interviews were conducted on-site at each participating health facility, during regular working hours. The head of each facility helped organise interview slots between patient consultations so that participation would not disrupt routine clinical activity. To minimise response and participation bias, the same standardised interview guide was used for every participant, administered consistently by data collectors, together with daily quality-control checks during the data collection period. Quality control procedures were implemented daily throughout the data collection period to ensure completeness, consistency, and accuracy.

## Sample size

The minimum sample size was calculated using Cochran’s formula adjusted for design effect due to multistage cluster sampling [13]:

$$no= DEFF\times\frac{Z\alpha^{2}\times p\times q}{d^{2}}$$

$${DEFF} = 1 + (m - 1)\times ICC$$

- *m* = average number of respondents per cluster = 4
- *ICC* = intraclass correlation coefficient = 0.008
- *Z*ₐ = 1.96 for 95% confidence
- *p* = assumed proportion using mRDTs = 0.80
- *q* = 1 − *p* = 0.20
- *d* = desired precision = 0.05

Clusters were defined as the randomly selected health facilities; each cluster comprised, on average, m = 4 HCWs, consistent with the design effect parameters used in the sample size formula. Facilities were randomly selected within each of the 9 district strata (first stage), and HCWs involved in malaria diagnosis were randomly selected within each selected facility (second stage), using a complete staff roster provided by each facility as the sampling frame.

This yielded a design effect (DEFF) of 1.024 and a required sample size of 252. After applying a finite population correction (target population ~ 1429) and adjusting for a 5% non-response rate, the minimum required sample size was 121. A total of 211 participants were ultimately enrolled.

### Definition of study variables

The primary outcomes of interest were general knowledge of malaria and mRDTs; attitudes regarding mRDTs reliability, ease of use, and equivalency; and reported practices in mRDTs use. Independent variables included professional category, health district, type of healthcare facility (public/private), years of experience, and additional training in infectious diseases or diagnostics.

Knowledge was assessed using a composite score derived from responses to five knowledge domains: definition of malaria, clinical symptoms, transmission routes, mRDTs operating principles, target antigens, testing procedures, and quality assurance steps. attitudes were assessed based on HCWs’ evaluations of mRDTs reliability, ease of use, brand equivalency, and availability. Practices were assessed based on reported frequency of use, clinical decision-making following positive or negative results, and adherence to national diagnostic guidelines. Scoring methods are detailed in supplementary table (S1 Table).

### Data analysis

Data were analysed using R statistical software (version 4.2.3). Continuous variables were described using the median and interquartile range [Q1, Q3], given their non-normal distribution as assessed by the Shapiro–Wilk test. Categorical variables were described as absolute and relative frequencies (n, %). Group comparisons for categorical variables were performed using the Chi-square test when expected frequencies exceeded 5, and Fisher’s exact test otherwise. The Kruskal–Wallis test was used to compare continuous variables “years of professional experience” across the knowledge, attitudes, and practice categorie*s*. A p-value < 0.05 was considered statistically significant.

Knowledge, attitudes, and practices were scored using predefined criteria. The scoring criteria followed a two-level hierarchical framework. First, each individual response component was coded using binary scoring (Yes = 1; No = 0), and cumulative scores were converted into percentages of the maximum attainable score for each question. These percentages were subsequently categorized as good (≥ 75%), intermediate (50–74.9%), or insufficient (< 50%). Second, for higher-order composite domains, these question-level categorical scores were transformed into ordinal values (good = 1, intermediate = 0.5, insufficient = 0) and aggregated to generate the final composite knowledge scores. Full operational details of the scoring procedure are provided in supplementary table (S1 Table).

## Results

### Characteristics of the study population

A total of 211 HCWs participated in the study. Nurses comprised the largest group (38%), followed by laboratory technicians (25%) and midwives (19%). Among the 09 health districts, Madibou and Ouenzé had the highest representation (25% and 12%, respectively). Most participants (74.4%) worked in public health facilities, and 81% reported no additional training in infectious diseases or diagnostics. The median duration of professional experience was 12 [3–18] years (Table 1).

**Table 1:** Sociodemographic characteristics of recruited participants

Variables	*n* (%)
Profession	
Health assistant	12 (5.7)
Nurse	80 (38.0)
Physician	10 (4.7)
Midwife	40 (19.0)
Laboratory technician	53 (25.0)
Others	16 (7.6)
Health district	
Bacongo	16 (7.5)
Djiri	23 (11.0)
Madibou	53 (25.0)
Makélékélé	21 (10.0)
Mfilou	20 (9.5)
Moungali	18 (8.5)
Ouenze	25 (12.0)
Poto-Poto	12 (5.7)
Talangaï	23 (11.0)
Type of structure	
Private health	54 (25.6)
Public health	157 (74.4)
Additional training in infectious disease or diagnosis	
No	171 (81.0)
Yes	40 (19.0)
Years of experience (years), median [Q1, Q3]	12 [3, 18]

### Knowledge of malaria and rapid diagnostic tests

Overall, only 9% of participants demonstrated good general knowledge of malaria, with most showing only intermediate understanding (67%). Most HCWs (63.5%) correctly defined malaria, but their knowledge of its symptoms was limited, while understanding of transmission modes was moderate.

Knowledge of mRDTs was generally poor. Very few participants understood the operating principles, target antigens, or key quality control steps. Detailed distributions are presented in Table 2.

**Table 2:** Healthcare workers knowledge on malaria and malaria rapid diagnostic tests

Knowledge	*n* (%)
General knowledge of malaria	
Good	19 (9.0)
Intermediate	142 (67.0)
Insufficient	50 (24.0)
Definition	
Good	134 (63.5)
Intermediate	68 (32.2)
Insufficient	9 (4.3)
Symptoms	
Good	24 (11.0)
Intermediate	56 (27.0)
Insufficient	131 (62.0)
Modes of transmission	
Good	17 (8.1)
Intermediate	194 (91.9)
Insufficient	0 (0.0)
Global knowledge about mRDT	
Good	0 (0.0)
Intermediate	20 (9.5)
Insufficient	191 (90.5)
Definition	
Good	46 (22.0)
Intermediate	68 (32.0)
Insufficient	97 (46.0)
How mRDT work	
Good	0 (0.0)
Intermediate	13 (6.2)
Insufficient	198 (93.8)
Antigens	
Good	16 (7.6)
Intermediate	13 (6.2)
Insufficient	182 (86.2)
Procedures	
Good	26 (12.3)
Intermediate	62 (29.4)
Insufficient	123 (58.3)
Critical steps to ensure accurate results	
Good	31 (15.0)
Intermediate	43 (20.0)
Insufficient	137 (65.0)

### Attitudes of healthcare workers regarding mRDTs

HCWs’ attitudes about mRDTs were mixed. While many (58.8%) considered them reliable due to their accuracy (45.2%) and speed (23.4%), others expressed doubts to false negatives (35.6%) and lack of precision (37.9%). Most HCWs (68.2%) agreed that not all available mRDTs are equally effective. The most preferred test was SD Bioline Malaria Ag Pf (70.8%), followed by SD Bioline Malaria Ag Pf (HRP2/pLDH) (16%). Accessibility of mRDTs was judged satisfactory by 91.5% of participants. A large majority (97%) found mRDTs easy to use, attributing this to simplicity (50.5%) and speed (43.6%). Additionally, 20.1% stated that no specialized training was needed (Table 3).

**Table 3:** Healthcare worker’s attitudes about malaria rapid diagnostic tests

Attitudes	*n*(%)
RDT is a reliable malaria diagnostic tool (Yes)	124 (58.8)
Reasons	
Speed	29 (23.4)
Simple	7 (5.6)
accuracy	56 (45.2)
quality	13 (10.5)
Compliance with procedures	6 (4.8)
No reviews	17 (13.7)
mRDT is a reliable malaria diagnostic tool (No)	87 (41.2)
Reasons	
False negative	31 (35.6)
False positive	4 (4.6)
No speed	1 (1.1)
Complex	6 (6.9)
Not accuracy	33 (37.9)
Bad quality	5 (5.7)
No reviews	9 (10.3)
mRDTs (non-equivalent effectiveness of mRDTs)	144 (68.2)
The most effective	
SD Bioline Malaria Ag Pf	102 (70.8)
SD Bioline Malaria Ag Pf (HRP2, pLDH)	23 (16.0)
First Response Malaria Ag Pf	4 (2.8)
ADV DX Malaria Pf	3 (2.1)
Malaria Pf.Pv	1 (0.7)
Don’t know	10 (7.0)
Accessibility and use of mRDTs in health centers	
Easy access to mRDTs in health facilities	193 (91.5)
mRDT is easy to use (Yes)	204 (97)
Reasons	
Speed	89 (43.6)
Simplicity	103 (50.5)
Without qualification	41 (20.1)
No Reviews	12 (5.9)
mRDT is Easy to use tool (No)	7 (3.3)
Reasons	
Complexity	3 (42.8)
Requires qualification	2 (28.6)
No Reviews	0 (0.0)

### Practices of healthcare workers in using mRDTs

Nearly all participants (96%) reported using mRDTs for malaria diagnosis, most commonly the SD Bioline Malaria Ag Pf test (53.5%). However, only about one-third (36%) had received specific training.

In practice, microscopy and mRDTs were often used together (86%), and over half of the respondents were unaware of the source of their test supplies.

Daily use of mRDTs was frequent (81.5%) of participants. Most performed the test when clinical symptoms were present (70.6%), though only 7.6% reported adherence to national malaria guidelines, and correct interpretation of mRDTs results was achieved by 62.5% (*n* = 132) of participants.

Following a positive mRDTs result, 73.9% prescribed antimalarial treatment, in case of a negative result, responses varied considerably (Table 4).

**Table 4:** Knowledge on the use of malaria rapid diagnostic tests

Practical	*n* (%)
Use of mRDTs for malaria diagnosis	203 (96.0)
SD Bioline Malaria Ag Pf	113 (53.5)
First Response Malaria Ag Pf	13 (6.1)
SD Bioline Malaria Ag Pf (*HRP2*,* pLDH*)	60 (28.4)
Paracheck Pf	5 (2.3)
ADV DX Malaria Pf	4 (1.8)
Malaria Pf.Pv	6 (2.8)
Training on the use of mRDTs	75 (36.0)
How is malaria diagnosed	
mRDT	15 (7.1)
Microscopy	15 (7.1)
Microscopy and mRDTs	181 (85.8)
mRDTs Suppliers	
Purchase (Pharmacies or others)	12 (5.7)
CAMEPS	47 (22.3)
CRS	11 (5.2)
PNLP	16 (7.6)
DGSSSa	24 (11.4)
No idea	112 (53.1)
Frequency of use of mRDTs	
Daily	172 (81.5)
Weekly	22 (10.4)
Monthly	17 (8.1)
Situation in which mRDTs is used	
National algorithm	16 (7.6)
Automatic	46 (21.8)
Symptoms	149 (70.6)
Interpretation of mRDTs results	
Good	132 (62.5)
Intermediate	17 (8.1)
Insufficient	62 (29.4)
Actions to take if mRDTs result is positive	
Treatment	156 (73.9)
Actions to take if mRDTs result is negative	
Consider other alternative diagnoses	56 (26.5)
Additional review	22 (10.4)
Microscopy	45 (21.3)
Symptomatologic treatment	38 (18.0)
Redo mRDTs	21 (9.9)
Action to take in case of discrepancy between mRDTs results and clinical symptoms	
Microscopy	83 (39.3)
Presumptive treatment	57 (27.0)
Additional examens (CRP, pregnancy test, CBC, )	62 (29.4)
Redo the mRDT	5 (2.4)
Refer to clinician	16 (7.6)
Don’t know	13 (6.2)

### Distribution of knowledge across professional and structural characteristics

#### General knowledge knowledge of malaria

Analysis of the data presented in S2 Table (supplementary table) revealed no statistically significant associations between HCWs’ general knowledge of malaria and any of the assessed variables (*p* > 0.05). Although nurses represented the largest share of those with good knowledge (36.8%), followed by laboratory technicians (21.0%), physicians were underrepresented (10.5%).

Geographically, no significant variations in knowledge levels were observed between districts (*p* = 0.454), although a notable proportion of participants with intermediate knowledge came from the Madibou district (28.9%). Regarding the type of healthcare facility, public institutions accounted for the majority (89.5%) of participants with good knowledge, but this was not statistically significant (*p* = 0.065). Neither years of professional experience (*p* = 0.4) nor additional training in infectious diseases or diagnostics (*p* = 0.295) was significantly associated with knowledge level.

### Knowledge of mRDT-targeted antigens

There was no significant association (*p* = 0.586) between professional category and knowledge of mRDT-targeted antigens S3 Table (supplementary table). Nurses and laboratory technicians had the highest representation among those with good knowledge (37.5%), followed by midwives (12.5%). No health assistants demonstrated good knowledge.

Professional experience (*p* = 0.596) and additional training in infectious diseases or diagnostics (*p* = 0.265) were not significantly associated with knowledge level, although 31.3% of those with good knowledge reported having a additional training.

### Knowledge of mRDTs procedures and quality assurance steps

None of the variables assessed including profession, health district, type of facility, years of experience, and additional training were significantly associated with knowledge of the mRDTs testing procedure (*p* > 0.05) S4 Table (supplementary table).

Nurses made up the majority of those with good knowledge (52.2%), followed by laboratory technicians (26.1%) and midwives (13.1%), while no physicians were represented in this category. Most participants with good knowledge came from the Djiri (30.5%) and Ouenzé (26.1%) districts, and from public facilities (65.2%).

Regarding knowledge of critical steps to ensure test accuracy, no significant association was found with profession (*p* = 0.312), years of experience (*p* = 0.546), or additional training (*p* = 0.523) S5 Table (supplementary table).

### Interpretation of mRDTs results

No significant association was observed between profession and ability to interpret mRDTs results (*p* = 0.637), though nurses were most frequently represented among those with poor interpretation skills. Geographic location (*p* = 0.401), years of experience (*p* = 0.418), and additional training (*p* = 0.087) also showed no significant relationship. However, HCWs in public facilities tended to interpret results more accurately than those in private settings (*p* = 0.061) S6 Table (supplementary table). 

### Attitudes of mRDTs efficacy and reliability by professional and structural characteristics.

#### Perceived efficacy equivalence of mRDTs

The professional category of HCWs was not significantly associated with Attitudes regarding the equivalence of mRDTs efficacy (*p* = 0.106). Among HCWs who believed that mRDTs brands were not equivalent in efficacy, nurses were the most likely to question the equivalence between different mRDTs brands (41.7%), while physicians were least likely to accept the idea of test equivalence (1.5%).

Similar trends were observed across health districts (*p* = 0.830), with HCWs from the Madibou district accounting for the largest share of those expressing scepticism (23.6%). Public sector HCWs more frequently believed in the equivalence of mRDTs (79.1%) compared to their counterparts in private facilities (20.9%), although this difference was not statistically significant (*p* = 0.313).

Neither years of professional experience (*p* > 0.9) nor additional training in infectious diseases or diagnostics (*p* = 0.600) was significantly associated with perceived efficacy equivalence of mRDTs S7 Table (supplementary table).

### Perceived reliability of mRDTs

There was no significant association between professional category and attitudes of mRDTs reliability (*p* = 0.349) S8 Table (supplementary table). Nurses constituted the largest group among those who perceived mRDTs as reliable (37.1%), followed by midwives (20.2%).

Across health districts, most of those who trusted mRDTs were from Madibou (21.8%), Djiri (14.5%), and Ouenzé (12.1%), but these differences were not statistically significant (*p* = 0.061). Similarly, HCWs in public facilities were more likely to report trust in mRDTs (76.6%) than those in private institutions, although this difference approached but did not reach statistical significance (*p* = 0.065).

No significant associations were found between perceived reliability and years of experience or additional training in infectious diseases or diagnostics.

## Discussion

This study provides a comprehensive assessment of HCWs’ knowledge, attitudes, and practices regarding the use of mRDTs across all 9 health districts of Brazzaville, Republic of Congo. The predominance of nurses among the participants reflects the healthcare system structure, where nurses serve as the primary providers of frontline care particularly in primary healthcare settings. Similar findings have been reported in other sub-Saharan African contexts [14, 15]. Despite the widespread use of mRDTs, this study highlights a persistent deficiency in malaria-related knowledge among HCWs. Many providers showed only a partial understanding of the disease’s transmission and clinical manifestations, revealing important gaps in training. These results align with previous studies from Africa showing that many HCWs lack sufficient knowledge about malaria, despite being actively involved in diagnosis and treatment [16, 17].

The findings concerning mRDTs knowledge are particularly concerning. None of the HCWs demonstrated good overall knowledge, and most were classified as having insufficient knowledge. This gap is critical, as appropriate knowledge is essential to ensure diagnostic accuracy, particularly in resource-limited environments where mRDTs are often the only feasible diagnostic tool [18, 19]. Inadequate knowledge can lead to poor diagnostic practices, the continuation of presumptive treatments contrary to WHO guidelines [1, 4, 20]. and even contribute to the emergence of antimalarial drug resistance [21, 22]. Moreover, lack of proficiency may erode community trust in health services, which is essential for the success of malaria control programs.

The attitudes of mRDTs among HCWs revealed a degree of uncertainty regarding their reliability. Although many participants expressed confidence in these tests, a substantial minority remained doubtful, often citing false-negative results or perceived lack of precision. Such scepticism has also been reported in studies from Nigeria and Kenya [11, 23–27], reflecting technical limitations of mRDTs, including reduced sensitivity at low parasitaemia and issues related to *pfhrp2* gene deletions [28–30]. Nevertheless, the prevalence of *pfhrp2* deletions in Brazzaville appears to be low [31]. Prior experience, training, and trust in diagnostic tools are known to shape these attitudes, as previously demonstrated by *Boyce et al. and Kyabayinze et al.* [32, 33].

A large proportion of HCWs believed that not all mRDTs brands are equally effective, with SD Bioline Malaria Ag Pf being the preferred option. This preference is consistent with previous performance evaluations showing superior accuracy of this brand [34–36]. Furthermore, the majority of participants found mRDTs easy to use citing simplicity and speed as key advantages. Similar attitudes have been reported in Nigeria, where ease of use was a critical factor in promoting test uptake [37].

However, despite this favourable attitude, the limited proportion of HCWs reported having received formal training on mRDTs use. This lack of training raises concerns about proper implementation and the risk of diagnostic errors due to procedural mistakes or misinterpretation [38, 39]. Moreover, very few participants reported following national malaria diagnostic guidelines. This suggests a gap either in guideline dissemination or in the ability of health systems to reinforce their implementation. These findings are consistent with previous research showing that compliance with guidelines is influenced by tool availability, work environment, and institutional support [40, 41].

When considering clinical practices following mRDTs results, the finding that 73.9% of HCWs prescribed antimalarial treatment after a positive mRDTs result is encouraging and consistent with WHO recommendations and reports from sub-Saharan Africa [42–44]. However, the 26.1% who did not follow test results remains a concern. This gap may reflect persistent doubts about test reliability, limited adherence to guidelines, or reliance on clinical judgment over laboratory confirmation. Similar patterns have been reported in other African settings [11, 23]. Overall, these results highlight that beyond availability of mRDTs, strengthening HCWs confidence in test results and improving adherence to guidelines through training and supervision is essential.

When faced with negative mRDTs results, HCWs demonstrated considerable variability in their clinical decision-making. Some tented to rely on alternative diagnoses or supplementary microscopy, while others resorted to symptomatic or presumptive treatment. This diversity of responses highlights the ongoing tension between test results and clinical judgement, particularly in contexts where confidence in diagnostic tools remains limited. Similar observations have been reported in Tanzania, where deviation from protocols was linked to perceived test unreliability [12]. The routine use of confirmatory microscopy following negative mRDTs, though sometimes necessary, also places a financial burden on the health system.

Interestingly, no significant associations were found between knowledge, attitude, or practices and professional or sociodemographic characteristics. This may reflect a systemic gap affecting the entire health workforce, rather than one limited to specific categories or regions. Similar uniformity has been reported in Tanzania [12], although it contrasts with findings from Nigeria, where additional training in infectious diseases was associated with better knowledge of mRDTs [11]. One strength of this study lies in its broad coverage, with representation from all health districts and a range of professional categories. This enhances the validity and generalizability of the findings. These data highlight the need for systematic and sustainable policy actions to strengthen malaria diagnostic practices, with direct implications for the National Malaria Control Programme in the Republic of Congo. In particular, the uniformly low levels of knowledge observed across all professional categories and health districts call for a comprehensive nationwide refresher training programme targeting all frontline HCWs, in both the public and private sectors. the implementation of structured and periodic refresher training programs on the use of mRDTs for healthcare professionals in both the public and private sectors is essential. Such training should emphasize the operating principles of mRDTs, correct interpretation of test results, adherence to national diagnostic guidelines, and appropriate management of patients with negative test results. The low rate of guideline compliance further suggests that dissemination alone is insufficient; active implementation support, strengthened supervision, and functional feedback mechanisms are required. Investment in these areas would be directly aligned with the objectives of the 2026–2030 National Malaria Control Strategy. This study presents several strengths as well as certain limitations that should be acknowledged. One of its main strengths lies in the comprehensive design, which included all health districts of Brazzaville and covered several primary healthcare facilities, with representation of different categories of healthcare professionals from both public and private facilities. This wide coverage enhances the generalizability of the findings. However, some limitations should be acknowledged. The use of self-reported data may have introduced social desirability bias, with participants potentially overestimating their knowledge or reporting ideal rather than actual practices.

## Conclusion

This study reveals significant deficiencies in the knowledge and practices related to mRDTs among HCWs in Brazzaville, Republic of Congo. Although the use of mRDTs is widespread, understanding of their principles, procedures, and targeted antigens remains alarmingly low. Furthermore, adherence to national malaria diagnostic guidelines is poor, with only a small fraction of HCWs applying the protocols correctly in routine practice.

These findings underscore the urgent need for comprehensive and continuous training programs targeting both urban and rural HCWs. Strengthening the dissemination and implementation of national guidelines, along with supervision and quality control mechanisms, is essential to improve diagnostic accuracy and the rational use of antimalarial treatments.

Addressing these systemic gaps will be critical to achieving effective malaria case management and supporting national efforts toward malaria control and elimination in the Republic of Congo.

Future research should explore the qualitative dimensions underlying the identified gaps, including healthcare workers’ motivations, institutional barriers, and health system constraints that influence diagnostic practices. Studies based on direct observation of practices rather than self-reported data, and including rural settings beyond the Brazzaville department, would provide a more comprehensive understanding of malaria diagnostic capacity in the Republic of Congo.

## Supplementary Information

Supplementary Material 1. Supplementary table: S1 Table: definition of scores; S2 Table. General knowledge about malaria according to healthcare worker characteristics; S3 Table: Knowledge antigens detected by mRDTs according to healthcare worker characteristics; S4 Table: Knowledge of testing procedures according to healthcare worker characteristics S5 Table: Knowledge of critical steps to ensure accurate malaria test results according to healthcare workers characteristics; S6 Table: Interpretation of mRDTs results according to healthcare workers characteristics; S7 Table: Perception about mRDTs equivalency of efficacity according to healthcare worker characteristics, and S8 Table: Perception about mRDTs reliability according to healthcare workers characteristics.

## Data Availability

The raw datasets generated and/ analyzed for this study will be made available by the authors on request.

## Abbreviations

CAMEPS: Centrale d’achat des Médicaments Essentiels et des Produits de Santé
DDSSA: Direction Générale des Services de la Santé
DEFF: Design effect
FCRM: Fondation Congolaise pour la Recherche Médicale
HCW: Healthcare Worker
mRDTs: Malaria Rapid Diagnosis Tests
PCR: polymerase chain reaction
PfHRP2: Plasmodium falciparum Histidine-Rich Protein 2
pLDH: Plasmodium lactate dehydrogenase

## Glossary

Knowledge: Level of theoretical understanding and technical competence possessed by healthcare workers regarding malaria diagnosis and the principles, use, and interpretation of mRDTs.
Attitudes: Beliefs, opinions, and evaluative judgments expressed by HCWs concerning the reliability, usefulness, and clinical relevance of mRDTs.
Practices: Self-reported or observed actions and clinical behaviors of HCWs related to the routine use, interpretation, and application of mRDTs in professional practice.
Willingness: The expressed readiness or intention of HCWs to adopt and use mRDTs in clinical decision-making, irrespective of external constraints such as institutional policies or test availability.
Geographic and logistical accessibility: Physical feasibility for the survey team to reach selected healthcare facilities during the data collection period, considering factors such as road conditions, seasonal constraints, and other logistical barriers
Accessibility of mRDTs for HCW: The effective availability and operational readiness of mRDTs kits within healthcare facilities, enabling their consistent and routine use in malaria diagnosis
