# Supplementary material for: Low practical knowledge but high willingness to use malaria rapid diagnostic tests among healthcare workers in the Republic of Congo
Source: BMC Public Health. 2026 Aug 11;26:2330. doi: 10.1186/s12889-026-28761-8 (PMC13459433; doi:10.1186/s12889-026-28761-8)
Supplement: Supplementary file 1 [file 12889_2026_28761_MOESM1_ESM.docx]

**S1 Table:** definition of scores

| **Question** | **Encode / Keywords** | **Odds** | **Score** | **Criteria** |
| --- | --- | --- | --- | --- |
| What is malaria? | Disease infection | Yes = 1; No = 0 | (Sum of odds/4) * 100 | Good ≥75%  Intermediate 50%-74.9%  Insufficient < 50% |
|  | Parasitic *Plasmodium* | Yes = 1; No = 0 |  |  |
|  | Bite | Yes = 1; No = 0 |  |  |
|  | Anopheles mosquito | Yes = 1; No = 0 |  |  |
| What are the main symptoms of malaria? | Fever | Yes = 1; No = 0 | (Sum of odds/8) *100 | Good ≥75%  Intermediate 50%-74.9%  Insufficient < 50% |
|  | Shiver | Yes = 1; No = 0 |  |  |
|  | Vomit | Yes = 1; No = 0 |  |  |
|  | Joint pain | Yes = 1; No = 0 |  |  |
|  | Muscle pain | Yes = 1; No = 0 |  |  |
|  | Headaches | Yes = 1; No = 0 |  |  |
|  | Asthenia | Yes = 1; No = 0 |  |  |
|  | Anorexia | Yes = 1; No = 0 |  |  |
| How is malaria transmitted? | Mosquito bite | Yes = 1; No = 0 | (Sum of odds /3) *100 | Good ≥75%  Intermediate 50%-74.9%  Insufficient < 50% |
|  | Transfusion Sanguine | Yes = 1; No = 0 |  |  |
|  | Transplacental transmission (mother - child) | Yes = 1; No = 0 |  |  |
| **General knowledge about malaria** | What is malaria? | Good = 1  Intermediate =0.5  Insufficient = 0 | (Sum of odds /3) *100 | Good ≥75%  Intermediate 50%-74.9%  Insufficient < 50% |
|  | What are the main symptoms of malaria? | Good = 1  Intermediate= 0.5  Insufficient = 0 |  |  |
|  | How is malaria transmitted? | Good = 1  Intermediate= 0.5  Insufficient = 0 |  |  |
| What is a rapid diagnostic test for malaria? | Test | Yes = 1; No = 0 | (Sum of odds /6) *100 | Good ≥75%  Intermediate 50%-74.9%  Insufficient < 50% |
|  | Antigen | Yes = 1; No = 0 |  |  |
|  | Malaria | Yes = 1; No = 0 |  |  |
|  | Fast | Yes = 1; No = 0 |  |  |
|  | Parasite | Yes = 1; No = 0 |  |  |
|  | Diagnostic Exam | Yes = 1; No = 0 |  |  |
| How does malaria RDT work? | Blood | Yes = 1; No = 0 | (Sum of odds /6) *100 | Good ≥75%  Intermediate 50%-74.9%  Insufficient < 50% |
|  | Membrane | Yes = 1; No = 0 |  |  |
|  | Antigen | Yes = 1; No = 0 |  |  |
|  | Antibody | Yes = 1; No = 0 |  |  |
|  | RDT cassette | Yes = 1; No = 0 |  |  |
|  | Line | Yes = 1; No = 0 |  |  |
| What antigens do malaria RDTs detect? | HRP2 | Yes = 1; No = 0 | (Sum of odds/3) *100 | Good = 3  Intermediate = 2  Insufficient = 0 or 1 |
|  | pLDH | Yes = 1; No = 0 |  |  |
|  | Aldolase | Yes = 1; No = 0 |  |  |
| Can you describe the procedure for performing malaria RDT? | Preparation of the equipment | Yes = 1; No = 0 | (Sum of odds /8) *100 | Good ≥75%  Intermediate 50%-74.9%  Insufficient < 50% |
|  | Wearing gloves | Yes = 1; No = 0 |  |  |
|  | Finger sanitization | Yes = 1; No = 0 |  |  |
|  | Finger prick | Yes = 1; No = 0 |  |  |
|  | Blood Collection | Yes = 1; No = 0 |  |  |
|  | Deposition of Blood | Yes = 1; No = 0 |  |  |
|  | Buffer | Yes = 1; No = 0 |  |  |
|  | Reading Time | Yes = 1; No = 0 |  |  |
| What are the critical steps to ensure accurate RDT results? | Diluent | Yes = 1; No = 0 | (Sum of odds /4) * 100 | Good ≥75%  Intermediate 50%-74.9%  Insufficient < 50% |
|  | Appropriate blood | Yes = 1; No = 0 |  |  |
|  | Reading time | Yes = 1; No = 0 |  |  |
|  | Product use no expires | Yes = 1; No = 0 |  |  |
| Critical steps to ensure accurate RDT results | Can you describe the procedure for performing malaria RDT? | Good = 1  Intermediate= 0.5  Insufficient = 0 | (Sum of odds /2) *100 | Good ≥75%  Intermediate 50%-74.9%  Insufficient < 50% |
|  | What are the critical steps to ensure accurate RDT results? | Good = 1  Intermediate= 0.5  Insufficient = 0 |  |  |
| **Knowledge of malaria RDTs** | What is a rapid diagnostic test for malaria? | Good = 1  Intermediate= 0.5  Insufficient = 0 | (Sum of odds /6) *100 | Good ≥75%  Intermediate 50%-74.9%  Insufficient < 50% |
|  | How does malaria RDT work? | Good = 1  Intermediate= 0.5  Insufficient = 0 |  |  |
|  | What antigens do malaria RDTs detect? | What antigens do malaria RDTs detect? |  |  |
|  | Can you describe the procedure for performing malaria RDT? | Good = 1  Intermediate= 0.5  Insufficient = 0 |  |  |
|  | What are the critical steps to ensure accurate RDT results? | Good = 1  Intermediate= 0.5  Insufficient = 0 |  |  |
|  | Critical steps to ensure accurate RDT results | Good = 1  Intermediate= 0.5  Insufficient = 0 |  |  |
| Interpretation of malaria RDT results | Test line | Yes = 1; No = 0 | (Sum of odds /5) * 100 | Good ≥75%  Intermediate 50%-74.9%  Insufficient < 50% |
|  | Control line | Yes = 1; No = 0 |  |  |
|  | Negative, positive or invalid test | Yes = 1; No = 0 |  |  |
|  | Absence of parasite | Yes = 1; No = 0 |  |  |
|  | Presence of parasite | Yes = 1; No = 0 |  |  |

**S2 Table:** General knowledge about malaria according to healthcare worker characteristics

| **Health professional characteristics** | **General knowledge about malaria** | | | |
| --- | --- | --- | --- | --- |
|  | **Good**  N = 19 | **Intermediate**  N = 142 | **Insufficient**  N=50 | **p-value** |
| **Profession** n (%) |  |  |  | 0.848 |
| Health Assistant | 1 (5.3) | 7 (4.9) | 4 (8.0) |  |
| Nurse | 7 (36.8) | 55 (38.7) | 18 (36.0) |  |
| Doctor | 2 (10.5) | 7 (4.9) | 1 (2.0) |  |
| Midwife | 3 (15.8) | 25 (17.6) | 12 (24.0) |  |
| Laboratory Technician | 4 (21.0) | 39 (27.5) | 10 (20.0) |  |
| Others (Pharmacy technician, ATS, Childcare worker, Physiotherapist) | 2 (10.5) | 9 (6.3) | 5 (10.0) |  |
| **Health District** n (%) |  |  |  | 0.454 |
| Bacongo | 1 (5.3) | 10 (7.0) | 5 (10.0) |  |
| Djiri | 1 (5.3) | 8 14 (9.9) | 8 (16.0) |  |
| Madibou | 3 (15.8) | 41 (28.9) | 9 (18.0) |  |
| Makélékélé | 4 (21) | 11 (7.7) | 6 (12.0) |  |
| Mfilou | 3 (15.8) | 12 (8.5) | 5 (10.0) |  |
| Moungali | 1 (5.3) | 13 (9.1) | 4 (8.0) |  |
| Ouenze | 1 (5.3) | 19 (13.4) | 5 (10.0) |  |
| Poto-Poto | 3 (15.8) | 6 (4.2) | 3 (6.0) |  |
| Talangaï | 2 (10.5) | 16 (11.3) | 5 (10.0) |  |
| **Types of structures** n (%) |  |  |  | 0.2822 |
| Private health (religious) | 2 (10.5) | 39 (27.5) | 13 (26.0) |  |
| Public health | 17 (89.5) | 103(72.5) | 37 (74) |  |
| **Years of experience** (years)  median [Q1, Q3] | 17 [4, 20] | 10 [3, 18] | 12 [3, 18] | 0.4 |
| **Specialization related to infectious diseases or diagnostics** n (%) |  |  |  | 0.295 |
| No | 17 (89.5) | 111(78.2) | 43 (86.0) |  |
| Yes | 2 (10.5) | 31 (21.8) | 7 (14.0) |  |

[Q1, Q3]: interquartile 25% and 75% respectively.

**S3 Table:** Knowledge antigens detected by mRDTs according to healthcare worker characteristics

| **Healthcare workers characteristics** | **knowledge of detected antigens** | | | |
| --- | --- | --- | --- | --- |
|  | **Good**  N = 16 | **Intermediate**  N = 13 | **Insufficient**  N=182 | **p-value** |
| **Profession** n (%) |  |  |  | 0.586 |
| Health Assistant | 0 (0) | 0 (0) | 12 (6.6) |  |
| Nurse | 6 (37.5) | 3 (23.1) | 71 (39) |  |
| Doctor | 1(6.2) | 1 (7.7) | 8 (4.4) |  |
| Midwife | 2 (12.5) | 3 (23.1) | 35 (19.3) |  |
| Laboratory Technician | 6 (37.5) | 6 (46.1) | 41 (22.5) |  |
| Others (Pharmacy technician, ATS, Childcare worker, Physiotherapist) | 1 (6.3) | 0 (0) | 15 (8.2) |  |
| **Years of experience** (years)  median [Q1, Q3] | 16 [3, 20] | 15 [10, 18] | 11 [3, 18] | 0.596 |
| **Specialization related to infectious diseases or diagnostics** n (%) |  |  |  | 0.265 |
| No | 11 (68.7) | 12 (92.3) | 148 (81.3) |  |
| Yes | 5 (31.3) | 1 (7.7) | 34 (18.7) |  |

[Q1, Q3]: interquartile 25% and 75% respectively.

**S4 Table:** Knowledge of testing procedures according to healthcare worker characteristics

| **Healthcare worker characteristics** | **Knowledge of testing procedures** | | | |
| --- | --- | --- | --- | --- |
|  | **Good**  N = 23 | **Intermediate**  N = 42 | **Insufficient**  N= 146 | **p-value** |
| **Profession** n (%) |  |  |  | 0.632 |
| Health Assistant | 1 (4.3) | 1 (2.4) | 10(6.8) |  |
| Nurse | 12 (52.2) | 15(35.7) | 53 (36.3) |  |
| Doctor | 0 (0.0) | 4 (9.5) | 6 (4.1) |  |
| Midwife | 3 (13.1) | 6 (14.3) | 31 (21.2) |  |
| Laboratory Technician | 6 (26.1) | 12 (28.6) | 35 (24.0) |  |
| Others (Pharmacy technician, ATS, Childcare worker,  Physiotherapist) | 1(4.3) | 4 (9.5) | 11 (7.5) |  |
| **Health District** n (%) |  |  |  | 0.454 |
| Bacongo | 0 (0.0) | 4 (9.6) | 12 (8.3) |  |
| Djiri | 7 (30.5) | 5 (11.9) | 11 (7.5) |  |
| Madibou | 0 (0.0) | 9 (21.5) | 44 (30.1) |  |
| Makélékélé | 2 (8.7) | 3 (7.1) | 16 (11.0) |  |
| Mfilou | 3 (13.0) | 3 (7.1) | 14 (9.6) |  |
| Moungali | 0 (0.0) | 6 (14.3) | 12 (8.2) |  |
| Ouenze | 6 (26.1) | 6 (14.3) | 13 (8.9) |  |
| Poto-Poto | 2 (8.7) | 3 (7.1) | 7 (4.8) |  |
| Talangaï | 3 (13.0) | 3 (7.1) | 17 (11.6) |  |
| **Types of structures** n (%) |  |  |  | 0.4 |
| Private (religious) | 8 (34.8) | 12 (7.6) | 34 (23.3) |  |
| Public | 15 (65.2) | 30 (71.4) | 112 (76.7) |  |
| **Years of experience** (years)  median [Q1, Q3] | 12 [6,18] | 10 [2, 18] | 12 [3, 18] | 0.4 |
| **Specialization related to infectious diseases or diagnostics** n (%) |  |  |  | 0.7 |
| No | 20 (87.0) | 35 (83.3) | 116 (79.4) |  |
| Yes | 3 (13.0) | 7 (16.7) | 30 (20.6) |  |

[Q1, Q3]: interquartile 25% and 75% respectively.

**S5 Table:** Knowledge of critical steps to ensure accurate malaria test results according to healthcare workers characteristics

| **Healthcare workers characteristics** | **Critical steps to ensure accurate results** | | | |
| --- | --- | --- | --- | --- |
|  | **Good**  N = 31 | **Intermediate**  N = 43 | **Insufficient**  N=137 | **p-value** |
| **Profession** n (%) |  |  |  | 0.312 |
| Health Assistant | 2 (6.4) | 0 (0) | 10 (7.3) |  |
| Nurse | 14 (45.2) | 20 (46.5) | 46 (33.6) |  |
| Doctor | 2 (6.4) | 3 (7) | 5 (3.6) |  |
| Midwife | 3 (9.7) | 5 (11.6) | 32 (23.4) |  |
| Laboratory Technician | 9 (29) | 12 (27.9) | 32 (23.4) |  |
| Others (Pharmacy technician, ATS, Childcare worker, Physiotherapist) | 1 (3.3) | 3 (7) | 12 (8.7) |  |
| **Years of experience** (years)  median [Q1, Q3] | 9 [3, 18] | 11 [3, 18] | 12 [3, 18] | 0.546 |
| **Specialization related to infectious diseases or diagnostics** n (%) |  |  |  | 0.523 |
| No | 26 (83.9) | 37 (86.1) | 108 (78.8) |  |
| Yes | 5 (16.1) | 6 (13.9) | 29 (21.2) |  |

[Q1, Q3]: interquartile 25% and 75% respectively.

**S6 Table:** Interpretation of mRDT results according to healthcare workers characteristics

| **Healthcare worker characteristics** | **Interpretation of mRDT results** | | | |
| --- | --- | --- | --- | --- |
|  | **Good**  N = 132 | **Intermediate**  N = 17 | **Insufficient**  N= 62 | **p-value** |
| **Profession** n (%) |  |  |  | 0.637 |
| Health Assistant | 7 (5.3) | 3 (17.6) | 2 (3.2) |  |
| Nurse | 51 (38.6) | 4 (23.6) | 25 (40.3) |  |
| Doctor | 7 (5.3) | 0 (0.0) | 3 (4.8) |  |
| Midwife | 24 (18.2) | 3 (17.6) | 13 (21.0) |  |
| Laboratory Technician | 34 (25.8) | 5 (29.4) | 14 (22.6) |  |
| Others (Pharmacy technician, ATS, Childcare worker,  Physiotherapist) | 9 (6.8) | 2 (11.8) | 5 (8.1) |  |
| **Health District** n (%) |  |  |  | 0.401 |
| Bacongo | 10 (7.6) | 3 (17.6) | 3 (4.8) |  |
| Djiri | 14 (10.6) | 0 (0.0) | 9 (14.5) |  |
| Madibou | 31 (23.5) | 3 (17.6) | 19 (30.6) |  |
| Makélékélé | 11 (8.3) | 2 (11.8) | 8 (12.9) |  |
| Mfilou | 17 (12.9) | 1 (5.9) | 2 (3.2) |  |
| Moungali | 10 (7.6) | 1 (5.9) | 7 (11.3) |  |
| Ouenze | 16 (12.1) | 3 (17.6) | 6 (9.7) |  |
| Poto-Poto | 6 (4.5) | 2 (11.8) | 4 (6.5) |  |
| Talangaï | 17 (12.9) | 2 (11.8) | 4 (6.5) |  |
| **Types of structures** n (%) |  |  |  | 0.061 |
| Private (religious) | 30 (22.7) | 5 (29.4) | 19 (30.7) |  |
| Public | 102(77.3) | 12 (70.6) | 43 (69.3) |  |
| **Years of experience** (years)  median [Q1, Q3] | 11 [3,18] | 16 [10, 19] | 10.5 [3, 19] | 0.418 |
| **Specialization related to infectious diseases or diagnostics** n (%) |  |  |  | 0.087 |
| No | 113(85.6) | 12 (70.6) | 46 (74.2) |  |
| Yes | 19 (14.4) | 5 (29.4) | 16 (25.8) |  |

**S7 Table:** Perception about mRDT equivalency of efficacity according to healthcare worker characteristics.

| **Healthcare worker characteristics** | **mRDTs have equivalent efficacy** | | **p-value** |
| --- | --- | --- | --- |
|  | **No**  **n=**144 | **Yes**  n=67 |  |
| **Occupation** n (%) |  |  | 0.106 |
| Health Assistant | 6 (4.2) | 6 (9.0) |  |
| Nurse | 60 (41.7) | 20 (29.8) |  |
| Doctor | 9 (6.3) | 1 (1.5) |  |
| Midwife | 28 (19.4) | 12 (17.9) |  |
| Laboratory Technician | 30 (20.8) | 23 (34.3) |  |
| Others (Pharmacy technician, ATS, Childcare worker,  Physiotherapist) | 11 (7.6) | 5 (7.5) |  |
| **Health District** n (%) |  |  | 0.83 |
| Bacongo | 10 (6.9) | 6 (9.0) |  |
| Djiri | 15 (10.5) | 8 (11.9) |  |
| Madibou | 34 (23.6) | 19 (28.4) |  |
| Makélékélé | 11 (7.6) | 10 (14.9) |  |
| Mfilou | 11 (7.6) | 9 (13.4) |  |
| Moungali | 16 (11.1) | 2 (3.0) |  |
| Ouenze | 21 (14.6) | 4 (6.0) |  |
| Poto-Poto | 11 (7.6) | 1 (1.5) |  |
| Talangaï | 15 (10.5) | 8 (11.9) |  |
| **Structure Type** n (%) |  |  | 0.313 |
| Private (religious) | 40 (27.8) | 14 (20.9) |  |
| Public | 104 (72.2) | 53 (79.1) |  |
| **Years of experience**, (years) median [Q1, Q3] | 12 [3, 18] | 10 [3, 18] | >0.9 |
| **Specialization on infectious diseases or diagnostics,** n (%) |  |  | 0.6 |
| No | 118 (81.9) | 53 (79.1) |  |
| Yes | 26 (18.1) | 14 (20.9) |  |

[Q1, Q3]: interquartile 25% and 75% respectively.

**S8 Table:** Perception about mRDT reliability according to healthcare workers characteristics

| **Healthcare worker characteristics** | **Perception about mRDT reliability** | | |
| --- | --- | --- | --- |
|  | **No**  n = 87 | **Yes**  n =124 | **p-value** |
| **Profession** n (%) |  |  | 0.349 |
| Health Assistant | 2 (2.3) | 10 (8.0) |  |
| Nurse | 34 (39.1) | 46 (37.1) |  |
| Doctor | 5 (5.7) | 5 (4.0) |  |
| Midwife | 15 (17.3) | 25 (20.2) |  |
| Laboratory Technician | 26 (29.9) | 27 (21.8) |  |
| Others (Pharmacy technician, ATS, Childcare worker, Physiotherapist) | 5 (5.7) | 11 (8.9) |  |
| **Health District** n (%) |  |  | 0.061 |
| Bacongo | 3 (3.4) | 13 (10.5) |  |
| Djiri | 5 (5.7) | 18 (14.5) |  |
| Madibou | 26 (30.0) | 27 (21.8) |  |
| Makélékélé | 8 (9.2) | 13 (10.5) |  |
| Mfilou | 8 (9.2) | 12 (9.7) |  |
| Moungali | 11 (12.6) | 7 (5.7) |  |
| Ouenze | 10 (11.5) | 15 (12.1) |  |
| Poto-Poto | 8 (9.2) | 4 (3.2) |  |
| Talangaï | 8 (9.2) | 15 (12.1) |  |
| **Types of structures** n (%) |  |  | 0.424 |
| Private (religious) | 25 (28.7) | 29 (23.4) |  |
| Public | 62 (71.3) | 95 (76.6) |  |
| **Years of experience** (years)  median [Q1, Q3] | 11 [2, 18] | 12 [3, 18] | 0.4 |
| **Specialization related to infectious diseases or diagnostics,** n (%) |  |  | 0.295 |
| No | 67 (77.0) | 104 (83.9) |  |
| Yes | 20 (23.0) | 20 (16.1) |  |

[Q1, Q3]: interquartile 25% and 75% respectively.
